# Concurrent Heat Waves and Extreme Ozone (O_3_) Episodes: Combined Atmospheric Patterns and Impact on Human Health

**DOI:** 10.3390/ijerph19052770

**Published:** 2022-02-27

**Authors:** Kenza Khomsi, Youssef Chelhaoui, Soukaina Alilou, Rania Souri, Houda Najmi, Zineb Souhaili

**Affiliations:** 1 General Directorate of Meteorology, B.P. 8106, Casablanca P.O. Box 5696, Morocco; youssefchelhaoui@gmail.com (Y.C.); najmi_houda@yahoo.fr (H.N.); 2 Laboratory of Chemistry-Biochemistry, Environment, Nutrition and Health, Faculty of Medicine and Pharmacy, Hassan II University, Ain Chock, Casablanca P.O. Box 5696, Morocco; z.souhaili@gmail.com; 3 International School of Public Health, Mohammed VI University for Health Sciences, Casablanca P.O. Box 5696, Morocco; 4 Department of Hydraulics Environment & Climate, Hassania School for Public Works, Casablanca P.O. Box 5696, Morocco; soukaina98alilou@gmail.com (S.A.); souri2rania@gmail.com (R.S.)

**Keywords:** heat wave, ozone episode, North Atlantic Oscillation, Mediterranean Oscillation, Saharan Oscillation, human health

## Abstract

More recurrent heat waves and extreme ozone (O_3_) episodes are likely to occur during the next decades and a key question is about the concurrence of those hazards, the atmospheric patterns behind their appearance, and their joint effect on human health. In this work, we use surface maximum temperature and O_3_ observations during extended summers in two cities from Morocco: Casablanca and Marrakech, between 2010 and 2019. We assess the connection between these data and climate indices (North Atlantic Oscillation (NAO), Mediterranean Oscillation (MO), and Saharan Oscillation (SaO)). We then identify concurrent heat waves and O_3_ episodes, the weather type behind this concurrence, and the combined health risks. Our findings show that the concurrence of heat waves and O_3_ episodes depends both on the specific city and the large-scale atmospheric circulation. The likely identified synoptic pattern is when the country is under the combined influence of an anticyclonic area in the north and the Saharan trough extending the depression centered in the south. This pattern generates a warm flow and may foster photochemical pollution. Our study is the first step toward the establishment of an alert system. It will help to provide recommendations for coping with concurrent heat waves and air pollution episodes.

## 1. Introduction

Industrial and traffic activities emit various pollutants that are harmful to human health. Ozone (O_3_) is among these air pollutants. O_3_ appears through a complex photochemical interaction triggered by sunlight and nitrogen oxides (NO_x_). NO_x_ can act as a sink or a source of O_3_ depending on their availability [1,2,3]. The total chemical balance is:

$${\mathrm{NO}}_{2}+\mathrm{hv}\;\to \mathrm{NO}+O$$

$$O+O_{2}\to O_{3}$$

$$\mathrm{NO}+O_{3}\to {\mathrm{NO}}_{2}+O_{2}$$

According to the study by Chiquetto and colleagues, the previously mentioned reactions may occur when higher air temperatures exceed 20 °C; the highest O_3_ mixing ratios appear under the warmest conditions [4]. Consequently, both NO_x_ emissions (the precursor) and the meteorological state control ambient O_3_ concentration. Temperature is the main meteorological factor to be directly involved in O_3_ extreme events [1,2]. Several documented studies have been carried out at national and international levels to assess these relationships. They have shown that ozone extremes frequently appear during heat waves and droughts and that this concurrence is partly due to specific synoptic patterns [5,6,7]. Jacob and Winner predicted that increased periods of air mass stagnation and increasing temperatures in the future would lead to increased O_3_ concentrations in many regions, particularly in polluted urban areas [8]. Similar results were found by Zhang et al. when they assessed the performance of chemistry climate models in simulating extremes of surface ozone, during 1990–2010, at northern midlatitudes [9]. In the Pearl River Delta region from China, Lin et al. studied the impact of local meteorological events on O_3_ spatiotemporal concentration. The authors used measured surface O_3_ concentration and meteorological parameters, during the extended summer (April–October), between 2006 and 2017. They have shown that O_3_ formation is triggered when temperatures exceed 33 °C. Hot events initiate extreme O_3_ events. Heat waves increase the O_3_ exceedance rate by 2.5 times [1]. More recently, in 2021, Wei et al. confirmed the triggering effect heat waves have on photochemical pollution and found that both phenomena may relate to atmospheric stagnation and blockage. This can promote their concurrence and amplify their impacts on health [10]. These results confirmed those by Tan et al. and Huang et al. studying air stagnation in Shanghai and China, respectively [11,12].

Over Europe, studies on heat wave episodes have consistently shown a synergistic effect of air pollution and high temperatures [5,13,14]. In 2007, Cristofanelli led research to evaluate the possible effects of heat wave phenomena on background O_3_ concentrations at a high mountain station in Italy. The authors observed unusually high O_3_ concentrations during the intense heat wave of August 2003. The station recorded the highest O_3_ concentrations when air masses originate from continental Europe and the Po basin boundary layer or the middle troposphere [15]. In 2008, Solberg et al. showed that the positive feedbacks between the weather conditions and O_3_ contributed to the elevated O_3_ [6]. More recently, in 2016, Otero and his team have assessed the relationship between local and synoptic meteorological conditions and surface O_3_ concentration in spring and summer over the period 1998–2012. They found that maximum temperature is a driver of maximum daily O_3_ and O_3_ extreme values, especially during warmer months. The study has also identified regions in Europe that may be particularly vulnerable to increased O_3_ episodes [16]. In Sydney, Australia, some studies showed that hot events and stronger near-surface temperature inversions limit the atmospheric mixing of locally emitted pollutants. They may worsen air quality levels in the city [2,17]. Heat waves in some cities from the same continent provoked occasional exceedances in O_3_ [18]. In the USA, the study by Zhang et al. established statistically significant higher ozone concentrations during extreme event periods. The authors compared ozone concentrations during extreme weather events and nonextreme events. In particular, they found that compound events yield high ozone formation that matches heat waves and atmospheric stagnation [19]. Schnell et al. reported that multiday and large-scale O_3_ pollution coincide with heat waves in different regions from eastern North America [7].

In Africa, the simultaneous appearance of heat waves and ozone episodes and their relationship with the large-scale atmospheric circulation is underconsidered by the scientific literature in the region. Khomsi et al. explored the concurrence of extreme O_3_ and hot events, in two urban cities from Morocco, during the extended summer (April–September) between 2009 and 2016 [20]. Authors reported that 33% of hot events matched O_3_ episodes in the coastal city of Casablanca, as compared to 70% in the inland city of Marrakech. They discussed the difference in results between Casablanca and Marrakech and highlighted the possible involvement of the general circulation in this disparity.

The authors recommended studying heat waves and photochemical pollution mechanisms. They suggested investigating the contribution of the general circulation to their concurrence. The purpose of the present study is to assess how extreme temperature may trigger high O_3_ levels and how this concurrence relates to large-scale circulation in Morocco. We discuss the combined impact of these events on human health and wellbeing. Our results will highlight some potential mechanisms responsible for heat waves and air pollution. They may lead to new insights in managing environmental extremes and their risk for public health.

**Figure 1:**
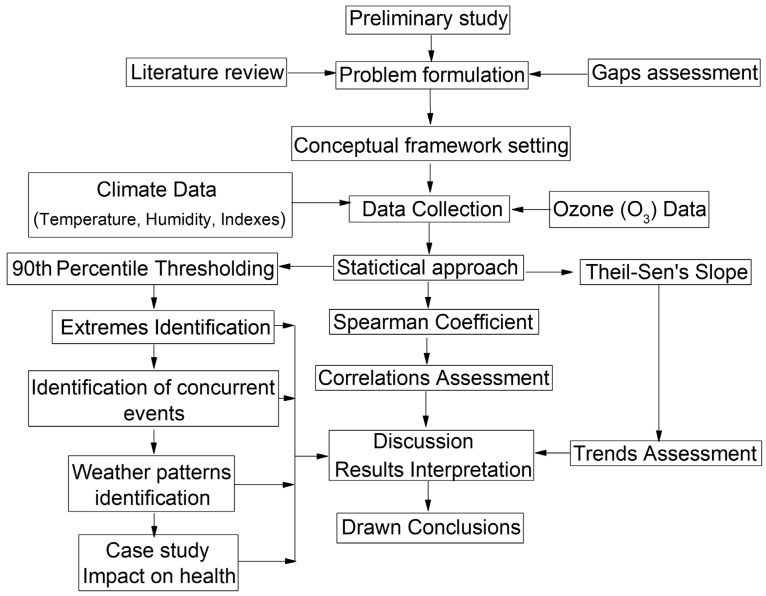
The research framework diagram.

Figure 1 introduces the research framework diagram for the current study. The preliminary study spotlights the need to explore compound events in Africa and Morocco, mainly in the context of climate change. The literature review highlights the importance of studying the concurrence of heat waves and ozone episodes and their link to the general circulation. Their combined effect on human health is an issue as well. Our formalized hypothesis is: “A particular large scale atmospheric pattern provokes heat waves that systemically trigger O_3_ episodes. Both extreme events are impacting human health simultaneously”. To explore this hypothesis, we have collected climate data (temperature, humidity, and climate indices) and O_3_ data. A percentile thresholding approach was applied to identify heat waves and extreme O_3_ episodes. Concomitant temperature and O_3_ events were then identified along with the large-scale synoptical pattern causing their concurrence. Moreover, we have assessed correlations between temperature and O_3_ time series and large-scale climate indexes. Finally, we have led a case study to evaluate the impact of a set of concomitant events on human health.

## 2. Materials and Methods

### 2.1. Study Area

**Figure 2:**
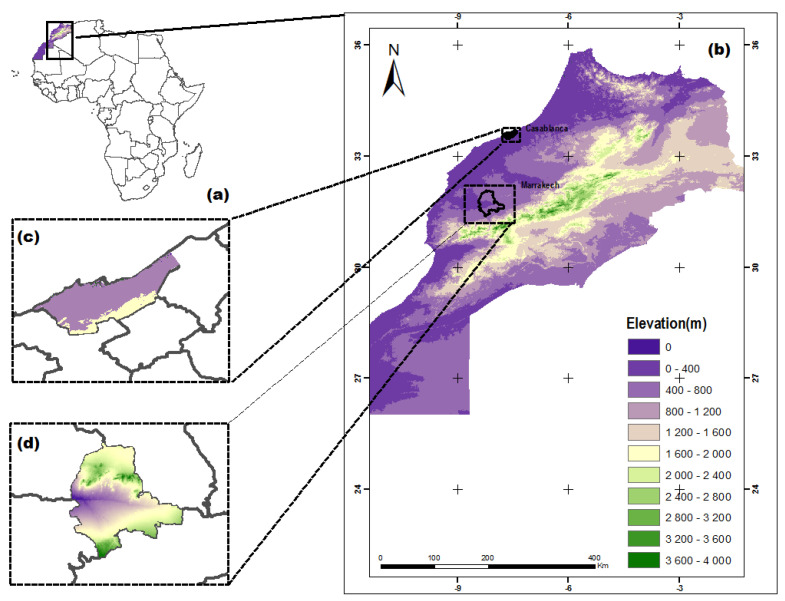
Location of the study area. Africa (**a**), North Morocco (**b**), Casablanca (**c**), and Marrakech (**d**).

Morocco is located in northwest Africa [20]. It is bordered by the Atlantic Ocean to the west, Algeria to the east, Mauritania to the south, and the Mediterranean Sea to the north (Figure 2).

Four mountain ranges dominate the country’s topography and divide it into three geographical regions: the mountainous interior including fertile plateaus and valleys; the Atlantic coastal lowlands; and the semiarid and arid areas of eastern and southern Morocco where the mountains gradually lie down into the Sahara Desert [21].

Casablanca and Marrakech (Figure 2) are two large urban cities in Morocco where serious pollution concerns may be met. Particularly, significant increase in the cities’ population rates was observed: 11% in Casablanca and 12% in Marrakech between 2004 and 2014. Casablanca is a coastal city and is the first most populous city in Morocco with more than 3,000,000 inhabitants and the highest rate of economic activities. Marrakech is an inland city and is the fourth largest city in the country with a population of over 900,000 inhabitants [22].

### 2.2. Data

#### 2.2.1. Temperature, Relative Humidity, and Ozone Data

In this study, we have used daily maximum temperature data, average relative humidity, and maximum O_3_. Data are recorded in Casablanca and Marrakech during the extended summer (April–September) between 2010 and 2019.

#### 2.2.2. Climate Indexes Data

A climate index characterizes an aspect of a geophysical system such as a circulation pattern. For this study, we have used three climate indexes: the North Atlantic Oscillation (NAO), the Mediterranean Oscillation (MO), and the Saharan Oscillation (SaO). The pressure centers for the NAO lie in the Atlantic Ocean. This connection consists of a north–south dipole of the sea level pressure (SLP) anomalies: one centered in Greenland and the other in the central North Atlantic [23]. The MO index represents a regional atmospheric circulation that characterizes the Mediterranean basin. It is a model of low frequency variability producing the opposition of barometric, thermal, and rainfall anomalies between the extremes of the Mediterranean basin. The MO Index is the difference in geopotential height anomalies between Algiers and Cairo [24]. We collected daily data of NAO and MO indexes during the study period from the Climatic Research Unit website (CRU, http://www.cru.uea.ac.uk/cru/data, accessed on 14 December 2021).

Khomsi et al. defined the SaO index. It characterizes the flow from the Saharan desert in the southern Morocco. The index refers to the difference between the normalized pressures at the Azores (37.79° N, −25.5° E) and Niamey (13.51° N, 2.10° E). For the aim of this work, we calculated the SaO index as follows (Equation (1)) [22]:

(1) $$SaOI_{d}=Pn_{d}\left(Açores\right)-Pn_{d}\left(Niamey\right)$$

$SaOI_{d}$: daily Saharan Oscillation Index;

$Pn_{d}$: daily normalized pressure during the study period.

We calculated the SaO index based on the sea level pressure data provided by the ERA5 reanalysis accessible in the Climate Data Store from the European Centre for Medium-Range Weather Forecasts (ECMWF) (CDS; https://cds.climate.copernicus.eu/#!/search?text=ERA5&type=dataset, accessed on 25 February 2022).

#### 2.2.3. Data Quality Assessment and Control

According to the World Meteorological Organization (WMO), QA refers to the process for maintaining a satisfactory level of quality in a data set or data collection so that the data available to potential users are sufficiently reliable and complete and can be used with confidence. QA is an end-to-end process that extends from the climate archive back through the data transmission channels to the point of observation. Providing feedback to observation providers and rectifying issues identified are also part of the QA process [25]. QC refers to the tools and practices employed to verify whether a reported data value is representative of what was intended to be measured and has not been contaminated by unrelated factors [25]. QC is the process of ensuring that errors in the data, or in the continuity of the data, are detected by checking the data to assess representativeness in time and space and internal consistency, and by flagging any potential errors or inconsistencies.

For the purpose of this study, climate and O_3_ observed data were provided by the General Directorate of Meteorology (GDM) in Morocco and are quality controlled and assessed before being available. At the GDM, meteorological data control is performed according to the recommendations of the WMO to National Meteorological and Hydrological Services [26,27,28]. To ensure data quality, the GDM adopts a clear process in the framework of its ISO 9001 certified management system. The process proactively prevents potential risks that could cause data quality deterioration, with a well-designed climate data management system and well-trained personnel aware of data quality culture. After collecting the data, data experts at the GDM identify and formulate data problems and implement corrective actions, through regular reviews and continuous improvement processes.

The control of air quality data, including O_3_, is performed according to the rules and recommendations from the Agency for the Environment and Energy Management [29]. The GDM adopts two main stages: (1) the automatic prevalidation that is a systemic process performed, according to special rules, at the level of the measuring device, the acquisition system, and the central concentration station; and (2) the expert validation by a qualified person according to two mandatory steps. The first step is the technical validation, which consists of checking the conformity of the whole measuring system. The second step is the environmental validation which consists of a cross-sectional investigation of the relevance and consistency of the obtained data.

To understand climate change and current weather extremes, it is important to have observations of the Earth system going back as far as possible in time. However, observations have always been unevenly distributed, and they come with errors [30]. Reanalyzes fill the gaps in the observational record, and they do so in a way that is consistent in time, thus minimizing any spurious signals of change [30]. The current study uses the ERA5 reanalysis data from the ECMWF. Within the Copernicus Climate Change Service (C3S), ECMWF is producing the ERA5 reanalysis, which provides the most complete picture currently possible of past weather and climate [30,31]. Prior to the production of the ERA5 reanalysis, work is carried out to improve the quality and availability of past observational data in terms of coverage and accuracy. Careful quality control is carried out as reanalyzes are produced, and their reliability is assessed by comparison with reanalyses produced at other institutes [30,31].

### 2.3. Methods

To identify yearly extreme events in temperature and ozone, we calculated the 90th percentiles for each year. This nonparametric approach studies the so-called soft extremes, i.e., rare events with a short return period (generally less than one year) [32]. The advantage of using these percentile thresholds for environmental change detection is their suitability to different climatic and environmental parameters. They allow easy comparison of trends between different regions, and they are easily understood and manageable for impact studies. The thresholding method is widely employed and recommended by the STARDEX (STAtistical and Regional dynamical Downscaling of EXtremes for European regions; https://crudata.uea.ac.uk/projects/stardex/, access on 14 December 2021) and the ETCCDI (Expert Team on Climate Change Detection and Indices) projects (Brown et al., 2010). Many studies in Morocco have used this approach [20,22,33].

For this study, we applied a thresholding approach to summer maximum temperature and ozone data between 2010 and 2019. We have used the following definitions as in the study by Khomsi et al. [20]:

- A hot event is a day that recorded maximum temperature greater than or equal to the 90th percentile;
- A heat wave is a succession of three hot events or more;
- An extreme ozone (O_3_) event is a day that recorded maximum O_3_ greater than or equal to the 90th percentile.

We analyzed the magnitudes of the trends in the studied time series using the nonparametric method proposed by Theil and Sen for univariate time series [34,35]. This approach involves computing slopes for all the pairs of ordinal time points and then using the median of these slopes as an estimate of the overall slope. Sen’s slope is robust against outliers, it is widely used for the estimation of trends in magnitudes of climate series [22,33,36,37]. The statistical significance of the trends is checked using the modified Mann–Kendall test proposed by Hamed and Rao [38] for autocorrelated time series. The test is performed at significance level of 5%.

The percentile thresholds calculated for maximum ozone data time series were compared to the thresholds stated by the Morocco national ambient air quality standards. The later sets O_3_ alert and information thresholds, respectively, to 200 μg·m^−3^ and 260 μg·m^−3^ for hourly averages.

Correlations between time series were estimated employing the Spearman coefficient. This statistical coefficient is used to measure the strength of the association between two variables and is widely used in climate studies [22,39].

Health impact of concurrent heat waves and O_3_ episodes were assessed through the evaluation of the related heat index (HI) and air quality health index (AQHI). HI as in Equation (2) was suggested by [40,41]. It is also known as the apparent temperature and is based upon assumptions about human physiology, behavior, clothing, and shade availability.

(2) $$\mathrm{HI}=-42.379+2.04901523T+10.14333127R-0.22475541\;T\times R-0.00683783T^{2}-0.05481\;$$

HI: Heat index (in degrees Celsius);

T: Ambient air temperature (in degrees Celsius)

R: Relative humidity (percentage value between 0 and 100)

Table 1 links HI values to the effects on human body.

AQHI in Equation (3) is an index that clarifies the impact of air quality on health. It provides advice to pay particular attention to people who are sensitive to air pollution [42].

(3) $$\mathrm{AQHI}=\left(\frac{1000}{10.4}\right)\times \left[\left(e^{0.000537\times O_{3}}-1\right)+\left(e^{0.000871\times {\mathrm{NO}}_{2}}-1\right)+\left(e^{0.000487\times {\mathrm{PM}}_{2.5}}-1\right)\right]$$

AQHI: Air quality health index;

O_3_: Average concentration of ozone (O_3_)

NO_2_: Average concentration of nitrogen dioxide

PM_2.5_: Average concentration of particles with a diameter of less than 2.5 µm (PM_2.5_)

Table 2 links AQHI to human health risk.

## 3. Results

### 3.1. Trends in Temperature and Ozone (O_3_) Extremes

Table 3 shows the trend magnitudes in yearly extreme temperature and O_3_, 90th percentiles of the datasets, and frequency of heat waves and O_3_ episodes. This data represents the study area during the summer seasons between 2010 and 2019. Trends in yearly average maximum temperature in Casablanca and Marrakech are negligible. 2015 and 2012 are the years that have recorded the highest temperatures in Casablanca (25.69 °C) and Marrakech (34.95 °C), respectively. 2018 has recorded the lowest temperature in both cities. Yearly maximum O_3_ is decreasing significantly in Casablanca and increasing in Marrakech. Trends in temperature percentiles in the cities of Casablanca and Marrakech are decreasing. Percentiles of maximum O_3_ are decreasing in Casablanca and increasing in Marrakech. None of the trends are statistically significant. O_3_ percentiles are still below the national thresholds for hourly averages. Extreme ozone episodes are slightly increasing in Marrakech; meanwhile, all the other trends are not statistically significant.

### 3.2. Concurrence of Heat Waves and Ozone Episodes (O_3_)

**Figure 3:**
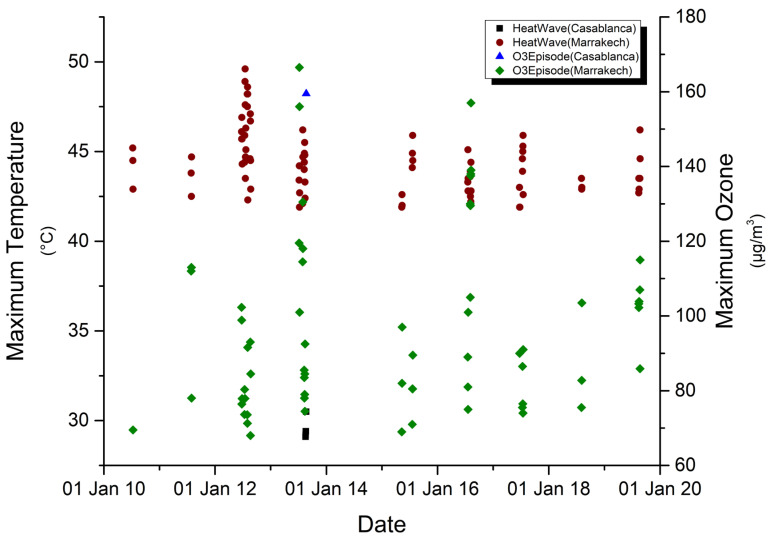
Concurrence between heat waves and ozone episodes during the summer season between 2010 and 2019.

The city of Casablanca has recorded 20 heat waves during the study period; only one heat wave is accompanied with an O_3_ extreme that also appeared in the city of Marrakech. Marrakech in turn has registered 26 heat waves, 14 of which was accompanied by O_3_ episodes. Figure 3 shows the concurrence between heat waves and O_3_ episodes. In many cases, O_3_ extremes match the first day of heat wave or appear slightly offset in time.

### 3.3. Heat Waves and Ozone Episodes (O_3_) Combiend Meteorological Patterns

**Figure 4:**
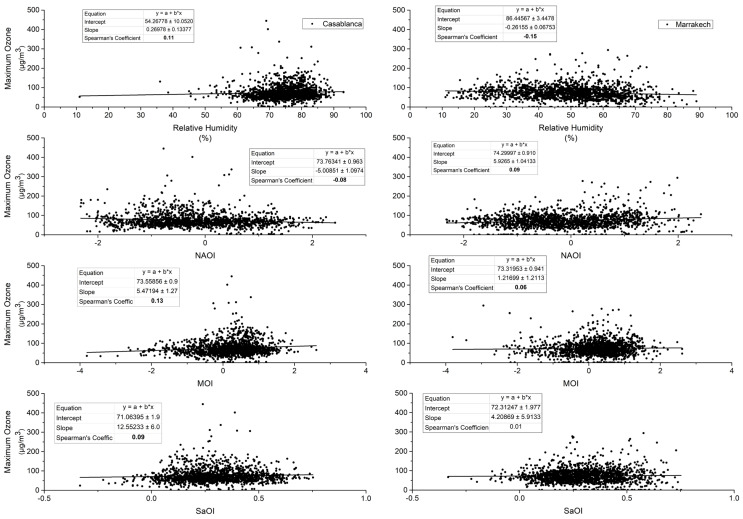
Correlation between extreme ozone, relative humidity, and climate indices during the summer season between 2010 and 2019. Spearman’s coefficient is significant when bold.

**Figure 5:**
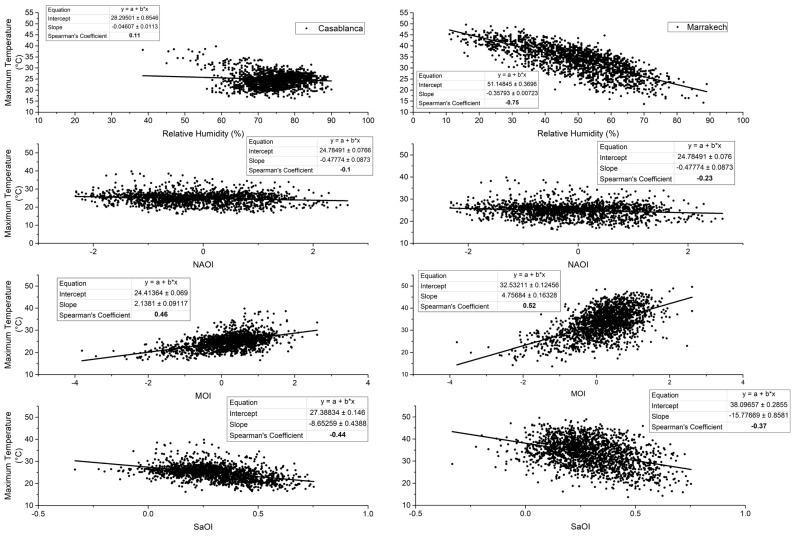
Correlation between maximum temperature, relative humidity, and climate indexes during the summer season between 2010 and 2019. Spearman’s coefficient is significant when bold.

Extreme episodes occur in Casablanca and Marrakech in different ways. This may be due to meteorological patterns and/or the geographical location as Casablanca is a coastal city and Marrakech is an inland. In this paragraph, we explore relationships between observed maximum temperature and O_3_ in Casablanca and Marrakech and relative humidity on the one hand, and between the same parameters and the above-defined climate indexes (NAO, MO, and SaO) on the other hand. Graphs and Spearman coefficients in Figure 4 and Figure 5 show that significant relationships, yet weak in many cases, exist between extreme O_3_, relative humidity and climate indexes in both cities.

Maximum O_3_ in Casablanca is negatively correlated with the NAO index and positively correlated with the remaining parameters. In Marrakech, the correlation between extreme O_3_ and relative humidity is negative and is positive with the other parameters. The correlation between maximum temperature and relative humidity in Marrakech is negative and quite strong. Positive, moderate, and significant correlations appear between maximum temperature and the MO index in both cities. Negative correlations of the same order appeared between maximum temperature and the SaO index.

**Figure 6:**
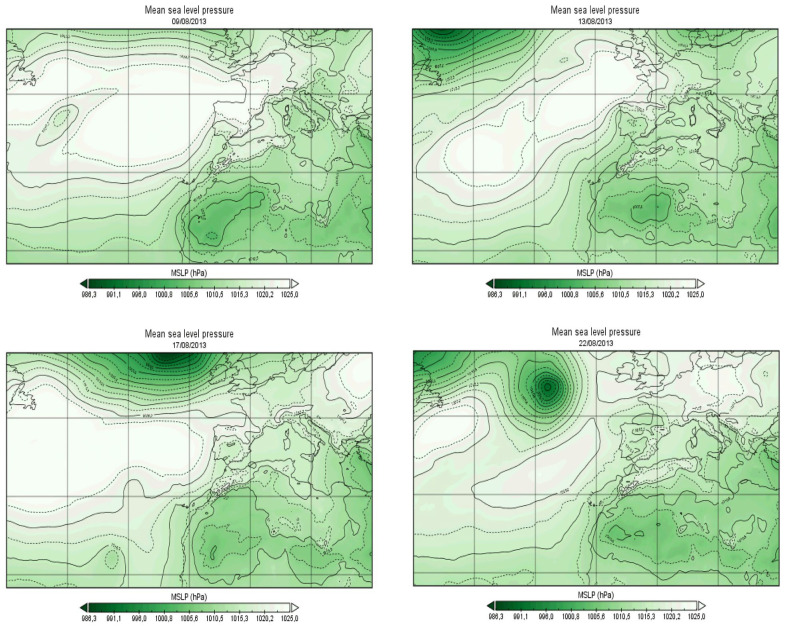
SLP fields for the 08–13–17–22/08/2013.

In parallel to this analysis, we redrew the SLP field for the commonly recorded heat wave and O_3_ episodes. We analyzed the flow impacting the study area on the large scale. This event lasts five days in Casablanca (9 August 2013 to 13 August 2013) and seven days in Marrakech (9 August 2013 to 15 August 2013). O_3_ episodes appeared slightly offset in time in Casablanca (17 August 2013 to 22 August 2013) and matched the same period in Marrakech (9 August 2013 to 13 August 2013). According to the SLP in Figure 6, the country is under the combined influence of the Azores High that spreads over the Atlantic and Western Europe, and the Saharan trough that extends the depression centered in the south. This trough invades the country, reaches the south of the European continent, and generates a warm southern flow over the region.

### 3.4. Impact of Concurrent Heat Waves and Ozone Episodes (O_3_) on Human Health

Table 4 shows HI and AQHI for the heat wave and O_3_ episode, recorded in Casablanca and Marrakech, from 9 August to 22 August 2013. Marrakech recorded more heat alerts than Casablanca. The hot days in Casablanca did not alert to any heat risk. However, the city has recorded one day with high-risk unhealthy air quality levels and five days with high risk. Marrakech recorded five days with combined extreme heat warning and a high risk of unhealthy air quality. This may have a joint impact on human respiratory health and thermal comfort.

## 4. Discussion

This study involved observed data in Casablanca and Marrakech (Morocco) during the extended summer (April–September) between 2010 and 2019. We analyzed trends in datasets and correlations with atmospheric indices. We then identified heat waves and O_3_ episodes and analyzed their concurrence. We also explored the atmospheric patterns behind this concurrence and the possible combined impacts on human health.

Taken together, our results suggest that, during the study period, no trends were recognized in average extreme temperature in both cities. This finding does not reinforce the general results of warmer trends in the country [33,43,44,45] and may be due to the short used study period or to the consideration of more recent data. Indeed, 2018 data was considered; this year has recorded the lowest temperature in both cities and was characterized with below normal winter temperatures and snowfall in the country [46]. This may have affected the expected warming trend. Extreme O_3_ is decreasing significantly in Casablanca and increasing in Marrakech. This may be due to the different geographical positions of the cities and the various local characteristics of outdoor pollution in each city. Casablanca is coastal; it plays a leading role in the economic development of Morocco. It hosts various industrial activities, an important automobile park, energy production and distribution, and the country’s largest ports and airport [47,48]. Considering its geographical position, Casablanca is still underexposed to sunlight, and even if it may register high NO_2_ concentration levels, the photochemical pollution is not its main feature. Moreover, Casablanca Tramway implementation in 2012 has played an important role in reducing NO_2_ emissions and then O_3_ generation. Marrakech, an inland city, hosts weak industrial activities and a rather important density of vehicles causing high NO_2_ concentrations levels. This makes the city a subject to photochemical pollution mainly due to its geographical location inducing strong sunlight. During spring and summer, O_3_ concentrations in the city reach alarming levels and exceed the thresholds [49,50]. Trends in temperature and O_3_ percentiles and extreme events echo the trends in averages. Extreme events may be partly explained by these averages. This statement is in complete agreement with many other climatological and air pollution studies over the area [20,33,43,51].

The concurrence of heat waves and O_3_ episodes in both cities was not systemic. Yet, when it happens, O_3_ episodes appear either in the first day of the heat wave or slightly offset in time. Marrakech recorded more concurring events than Casablanca. This spotlights the role of the geographical location of the cities and the influence of meteorological parameters on events’ occurrence. This influence was highlighted in many previous studies as well [1,6,7,9,10,11,15,16,19,20,52]. For example, the study by Chen et al. concluded that soaring ozone concentrations across China in 2017 could be mainly attributed to the notable change of meteorological conditions in 2017, characterized with rising temperature and sunshine duration and decreasing relative humidity [52]. In eastern North America, Schnell et al. mentioned that although heat waves and air pollution episodes of O_3_ and fine particles are not exactly co-occurring, many of these extremes show connectedness with consistent offsets in space and in time and clearly coincide with large-scale meteorological features [7]. Wei et al. mentioned similar results in a more recent study. Authors concluded that an increase in extreme air stagnation and hot days led to the worsening of regional O_3_ pollution events in different regions from China [10]. These different findings explain the correlations between extreme O_3_ and relative humidity in Casablanca (positive) and Marrakech (negative) and clarifies the strong negative correlation between maximum temperature and relative humidity in Marrakech compared to Casablanca.

Positive, moderate, and significant correlations appear between maximum temperature and MO index in both cities. Negative correlations of the same order appear with the SaO index. This finding recalls results from other studies [22,33]. In 2016, Khomsi et al. confirmed that summer average maximum temperature is affected by the MO in Marrakech [33]. In 2020, the same author and his team elucidated the relationship between the MO and the average concentrations of particulate matter 10 μm or less in diameter (PM_10_) and confirms that MO and SaO are affecting the particulate pollution oppositely [22]. The northeasterly to southwesterly continental warm flow that is triggered by the Saharan trough and influenced by the high-pressure area in the north causes the temperature to increase and foster particulate pollution. If extended, the high-pressure area in the north of Morocco can create a blocking situation and induce photochemical pollution as well. We expected stronger correlations between maximum O_3_, relative humidity, and climate indexes. The found weak links may be due to the local features of photochemical pollution in both cities and the continuous supply of local primary pollutants (NO_x_ for example) from the large vehicle fleet in both cities or from industrial activities in Casablanca. Moreover, this study was conducted in the extended summer when sunshine duration is the main factor responsible of O_3_ generation.

The case study of the heat wave from 9 August 2013 to 22 August 2013 confirmed the above findings. O_3_ episodes appear slightly offset in time in Casablanca and in the same period in Marrakech. The country was under the combined influence of the Azores High, spreading over the Atlantic and the Western Europe, and the Saharan trough extending the depression centered in the south. This trough invades the country, reaches the south of the European continent. and generates a warm southern flow over the region. This synoptic pattern explains the correlations between maximum temperatures and MO and SaO indexes and explicates the role of the anticyclonic area over the north of Morocco in trapping the warm air over the country or allowing it to attend the European continent. During the previously mentioned case study, the combined risk on human health and thermal comfort was registered mainly in Marrakech. Coastal temperature and relative humidity in the coastal city of Casablanca seems to reduce the perception of heat risk, yet a high risk of unhealthy air quality levels was registered. If available, health data can help in further developing this aspect.

The analysis in this study examines heat waves and O_3_ episodes in Casablanca and Marrakech, and therefore, the results are limited to these regions that have their own geographical locations and climate conditions. Therefore, our findings could not be generalized to a larger area. Our results are also limited to the available data, the study period, and the methods used, especially to identify extreme events. In climatology, a ten-year period is not sufficient enough to figure out climate trends and variations. Further studies are worth conducting when more data are available, to cover more regions, using more developed methods such as machine learning analysis, and including other atmospheric indices such as ENSO or SaO in different pressure levels or extending the temporal coverage in the future. Moreover, our study considers O_3_ anthropogenic sources involving mainly NO_X_ as a sink or a source. The study does not consider the reduced ozone removal by water-stressed vegetation that can exacerbate ozone air pollution and worsen peak ozone episodes [5]. Health data were not available to this study to conduct a deep assessment of health impact, as such heat- and air-pollution-related mortality and morbidity data are worth considering when available to study the combined impact of heat waves and air pollution episodes on human health and well-being in depth.

## 5. Conclusions

This work has focused on exploring the concurrence of heat waves and O_3_ episodes in two cities from Morocco (Casablanca and Marrakech) during the summer season between 2010 and 2019. It studies the relationship of this type of extremes with atmospheric circulation indexes and their combined effect on human health and well-being.

The research confirms that the concurrence of heat waves and ozone episodes depends both on the specific city—hence, local sources—and on large-scale atmospheric circulation—thus, meteorological parameters. However, it does not support the simple mechanistic argument that warmer temperatures make ozone pollution more severe. Moreover, this study identifies the synoptic pattern likely behind the occurrence of these extreme events. This pattern and related meteorological factors can be linked to direct health effects. When more data become available, the contribution from local and global air pollution sources may be investigated. This emphasizes the need for more local to regional studies. It would be worthwhile then to lead such a study for other regions in Morocco and consider other pollutants. Obtained results could be compared with those from the present study.

Although many previous studies have examined air pollution in Casablanca and Marrakech, to the best of our knowledge, our work is the first attempt to assess combined features inducing large-scale atmospheric circulation and health effects. Our research explores the hypothesis that particular weather patterns increase the vulnerability of individuals, especially those sensitive to air pollution. The current work and similar additional studies may help the establishment of a climate/air/health alert system and provide recommendations for implementing plans and strategies to cope with concurrent heat waves and air pollution episodes. Such strategies would contribute to enhance health services capacities in dealing with the combined and brusque impact of environmental extremes on public health.

## Figures and Tables

**Table 1:** Heat Index and impact on human comfort [40,41].

Temperature (°C)	Impact on Human Comfort
27–32 °C	Caution fatigue is possible with prolonged exposure and activity. Continuing activity could result heat cramps.
32–41 °C	Extreme caution: heat cramps and heat exhaustion are possible. Continuing activity could result in heat stroke.
41–54 °C	Danger: heat cramps and heat exhaustion are likely; heat stroke is probable with continued activity.
Over 54 °C	Extreme danger: heat stroke is imminent.

**Table 2:** Air Quality Health Index and health risk [42].

AQHI	Health Risk
1–3	Low Risk
4–6	Moderate Risk
7–10	High Risk
Above 10	Very High Risk

**Table 3:** Trends in temperature and O_3_ extremes.

Theil-Sen’s Slope	Casablanca	Marrakech
Yearly Tmax (°C/decade)	−0.08	0.04
Yearly O_3_max (µg·m^−3^/decade)	**−4.38**	5.78
Tmax percentile °C/decade)	−0.11	−4.23
O_3_max percentile (µg·m^−3^/decade)	−0.21	7.64
Heat waves (heat wave/decade)	−1.25	−0.25
O_3_ episodes (episode/decade)	0	**0.8**

Bold character: Result is statistically significant.

**Table 4:** Heat and air quality health indices between 9 August 2013 and 22 August 2013 in Casablanca and Marrakech.

Days of the Episode	Casablanca		Marrakech	
	Heat Risk	Air Quality Risk	Heat Risk	Air Quality Risk
9 August 2013	No Risk	High Risk	Extreme Caution	High Risk
10 August 2013	No Risk	High Risk	Extreme Caution	High Risk
11 August 2013	No Risk	Very High Risk	Extreme Caution	Moderate Risk
12 August 2013	No Risk	High Risk	Extreme Caution	Moderate Risk
13 August 2013	No Risk	Moderate Risk	Extreme Caution	High Risk
14 August 2013	No Risk	Moderate Risk	Extreme Caution	Moderate Risk
15 August 2013	No Risk	Moderate Risk	Extreme Caution	High Risk
15 August 2013	No Risk	Moderate Risk	Caution	High Risk
17 August 2013	No Risk	Moderate Risk	No Risk	Moderate Risk
18 August 2013	No Risk	Moderate Risk	Caution	High Risk
19 August 2013	No Risk	High Risk	Caution	High Risk
19 August 2013	No Risk	High Risk	Extreme Caution	High Risk
21 August 2013	No Risk	Moderate Risk	Caution	High Risk
22 August 2013	No Risk	Moderate Risk	Caution	Moderate Risk

## Data Availability

For the purpose of this study. We collected observed air quality and weather data from the General Directorate of Meteorology. These data are not publicly available and the providing organization should be contacted for any request in this respect. Data related to climate indexes NAO and MO are available on the Climatic Research Unit website (CRU, https://crudata.uea.ac.uk/cru/data/nao/ and https://crudata.uea.ac.uk/cru/data/moi/, access on 14 December 2021). Sea Level Pressure data that serve the calculation of the SO index are provided by the ERA5 reanalysis accessible at the Climate Data Store from the European Centre for Medium-Range Weather Forecasts (ECMWF) (CDS; https://cds.climate.copernicus.eu/#!/search?text=ERA5&type=dataset, access on 14 December 2021).
